# 3,4-Methylenedioxy methamphetamine, synthetic cathinones and psychedelics: From recreational to novel psychotherapeutic drugs

**DOI:** 10.3389/fpsyt.2022.990405

**Published:** 2022-10-03

**Authors:** Raúl López-Arnau, Jordi Camarasa, Marcel·lí Carbó, Núria Nadal-Gratacós, Pol Puigseslloses, María Espinosa-Velasco, Edurne Urquizu, Elena Escubedo, David Pubill

**Affiliations:** ^1^Pharmacology Section, Department of Pharmacology, Toxicology and Therapeutic Chemistry, Facultat de Farmàcia i Ciències de l’Alimentació, Universitat de Barcelona, Barcelona, Spain; ^2^Institut de Biomedicina de la Universitat de Barcelona (IBUB), Barcelona, Spain; ^3^Pharmaceutical Chemistry Group (GQF), IQS School of Engineering, Universitat Ramon Llull, Barcelona, Spain

**Keywords:** antidepressant (AD), cathinone, LSD, MDMA, NPS, psychedelics, psilocybin, tryptamine

## Abstract

The utility of classical drugs used to treat psychiatric disorders (e.g., antidepressants, anxiolytics) is often limited by issues of lack of efficacy, delayed onset of action or side effects. Psychoactive substances have a long history of being used as tools to alter consciousness and as a gateway to approach the unknown and the divinities. These substances were initially obtained from plants and animals and more recently by chemical synthesis, and its consumption evolved toward a more recreational use, leading to drug abuse-related disorders, trafficking, and subsequent banning by the authorities. However, these substances, by modulation of certain neurochemical pathways, have been proven to have a beneficial effect on some psychiatric disorders. This evidence obtained under medically controlled conditions and often associated with psychotherapy, makes these substances an alternative to conventional medicines, to which in many cases the patient does not respond properly. Such disorders include post-traumatic stress disease and treatment-resistant depression, for which classical drugs such as MDMA, ketamine, psilocybin and LSD, among others, have already been clinically tested, reporting successful outcomes. The irruption of new psychoactive substances (NPS), especially during the last decade and despite their recreational and illicit uses, has enlarged the library of substances with potential utility on these disorders. In fact, many of them were synthetized with therapeutic purposes and were withdrawn for concrete reasons (e.g., adverse effects, improper pharmacological profile). In this review we focus on the basis, existing evidence and possible use of synthetic cathinones and psychedelics (specially tryptamines) for the treatment of mental illnesses and the properties that should be found in NPS to obtain new therapeutic compounds.

## Introduction

Psychiatric are very prevalent medical conditions nowadays. These constitute a public health concern as they often lead to disability, with a high associated sanitary cost. Among these disorders, it is estimated that between 1.0 and 16.9% of the world population suffer from depression (1) and around 3.8–25% have anxiety (2). The number of incident cases of depression worldwide increased by 49.9% from 1990 to 2017 (3) and it is also a major risk factor for suicide. Although there are several clinically tested and authorized medicines and protocols to treat these illnesses, about 50% of patients suffering from major depressive disorder receiving treatment fail to respond (4). When patients fail to respond to two or more antidepressants, it is considered that they suffer from treatment-resistant depression (5).

In addition, patients who respond to classic antidepressant treatment suffer from undesirable and frequent side effects, and many discontinue treatment (6). Furthermore, due to their late-onset clinical effect (7), these medications are not an ideal treatment for certain cases of anxiety and depression, so alternative treatments are highly sought after. Moreover, from the beginning of this century, fear-related disorders such post-traumatic stress disorder (PTSD) and specific phobias have also raised their prevalence. These disorders are also accompanied by a decrease in quality of life and by severe depression and anxiety (8, 9).

**FIGURE 1:**
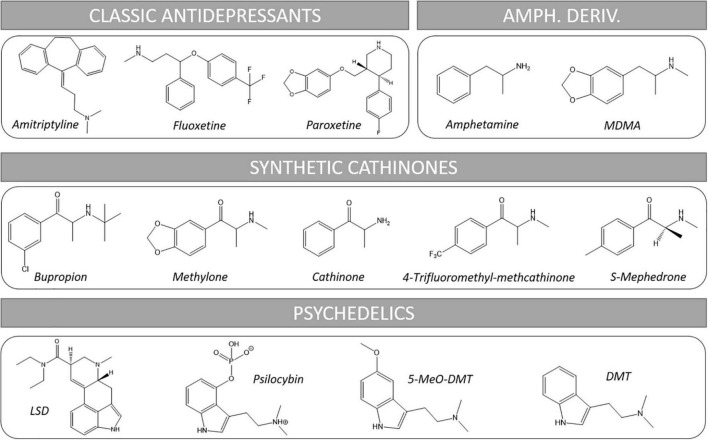
Chemical structures of representative substances cited in this review. Amph. Deriv., amphetamine derivatives.

Psychedelic substances have been used for millennia since the dawn of humanity for magic-religious rituals to communicate with divinities, the spiritual world of the deceased and to obtain knowledge and healing (10). These substances were consumed from vegetal and animal preparations. Classical examples of these drugs are *ayahuasca*, *peyote* and *magic mushrooms.* Psychedelic effects include an altered state of consciousness characterized by distortions of perception, hallucinations, visions, dissolution of self -boundaries and “the experience of union with the world” (11). During the 20th century, the advances in chemistry led to the isolation and structural elucidation of the active molecules of these natural drugs and to the synthesis of a great number of analogs. Such structures included tryptamines (*N*,*N*-dimethyltryptamine (DMT), 5-methoxy-*N*,*N*-dimethyltryptamine (5-MeO-DMT), psilocybin) and indole alkylamines (e.g., lysergic acid diethylamide, LSD). On the other hand, a great number of amphetamine derivatives was synthetized due to their vasomotor, psychostimulant, and anorectic effects, from which we highlight 3,4-methylenedioxymethamphetamine (MDMA) (see Figure 1 for representative chemical structures).

Many of these substances, such as LSD, were tested for possible medical uses during the 1950s and 1960s, demonstrating that they could be used to treat anxiety and depression (12). However, as some of them spread into the illicit recreational market, the Food and Drug Administration (FDA) banned any pharmacological research into them in 1970 by classifying these substances in Schedule I, the most restrictive category of drugs (13). Similarly, for MDMA, uncontrolled published reports suggested that, when administered in conjunction with psychotherapy, it could provide substantial benefits for those afflicted with a variety of mental disorders and so was used in the United States from 1977 to 1985, when it became scheduled under the Controlled Substances Act (CSA) (14).

However, the need of alternatives to clinical drugs and based on such previous evidence fueled efforts to re-take clinical studies with these scheduled substances, although most of them were denied regulatory approval. Thus, clinical trials with MDMA and several psychedelics such as LSD, psilocybin and DMT on depression, anxiety and PTSD have been carried out with promising results, even in treatment-resistant cases (15, 16). Such studies demonstrate the psychotherapeutic utility of these compounds and encourage to carry on with these studies but, as it also happens with clinically approved medicines, these drugs may have side effects, contraindications and some non-optimal pharmaceutical properties that claim for the search of alternatives which, retaining the therapeutic activity, overcome these issues.

Consequently, several associations raised to promote the research on the clinical use of psychedelic drugs to enlarge the therapeutic arsenal to treat several mood disorders. These include the Multidisciplinary Association for Psychedelic Studies (MAPS),^1^ a non-profit organization that was founded in 1986 to increase the knowledge base of psychedelic substances; the Heffter Research Institute, a non-profit organization founded in 1993 that promotes research with classic hallucinogens and psychedelics, predominantly psilocybin; and the Beckley Foundation, which promotes the use of psilocybin for depression and LSD for pain.

The irruption of new psychoactive substances (NPS), especially during the last decade and despite their recreational and illicit uses, has enlarged the library of substances with potential utility on these disorders. In fact, many of them were initially synthetized with therapeutical purposes and were withdrawn for concrete reasons that did not make these substances appropriate for the purposed medical use (e.g., adverse effects, improper pharmacological profile, etc.). NPS include a range of chemical structures which intend to act as replacements to classical banned substances (e.g., cocaine, MDMA, LSD, cannabis, opioids, etc.), so that they can be initially freely sold in presentations “not intended for human use” such as bath salts, plant food, incense, research chemicals, etc. (17). Moreover, as they are classified and banned, they are replaced by a new generation of non-controlled molecules enlarging the library of compounds that might, under a clinical setting, be used in therapeutics. The possibilities of therapeutic uses of synthetic cathinones and synthetic cannabinoids have been cited and summarized in a recent review (18), which also warns about the undesirable effects of these substances, which may preclude such therapeutic uses.

In this review we will focus in two groups of NPS, namely synthetic cathinones and psychedelics, as they share at least part of their mechanism of action with psychotherapeutic drugs (e.g., antidepressants) and may provide additional actions that contribute to the therapeutic effect. In fact, there is already evidence of clinical trials with some of these substances. We will discuss the pharmacodynamic requirements, review the previous evidence and the properties expected in a new drug to become useful in therapeutics of mood disorders.

## Mechanisms of action involved in the antidepressant effect

### Monoamine enhancers

Nowadays, depression and several anxiety disorders are usually treated with antidepressants (AD) (see Figure 1 for representative chemical structures). These include from classical tricyclic antidepressants (TCAs, e.g., amitriptyline) to the more recent selective serotonin (5-HT) reuptake inhibitors (SSRIs, e.g., fluoxetine, paroxetine, sertraline, escitalopram…) and atypical antidepressants which combine several mechanisms of action including inhibition of some monoamine transporter/s plus effects on concrete receptors such as histaminergic or serotonergic (e.g., trazodone, mianserin, mirtazapine, etc.) (19–22).

The main mechanism of action of these drugs is the enhancement of monoamine neurotransmission in the CNS, mainly of norepinephrine (NE), 5-HT and dopamine (DA), by interacting with presynaptic transporters and inhibiting their reuptake, thus restoring the hypothetically reduced neurotransmitter levels in certain brain areas (7). The increased neurotransmitter levels result in activation of receptors whose signaling pathways contribute to the antidepressant effect. As their name indicates, SSRIs, currently the most used AD, possess much higher selectivity for the 5-HT transporter (SERT) than for the transporters of norepinephrine (NET) and dopamine (DAT), and were developed after the serotonergic theory of depression postulated by Lapin and Oxenkrug (23) which suggests that serotonergic pathways play a major role in depression.

Not devoid but with milder adverse effects than TCAs, SSRIs rapidly reached a prevalent place in the treatment of mood disorders, although they can interfere with libido and appetite, cause vomiting and produce nausea, irritability, anxiety, insomnia and headaches. Also, and to a lesser extent, they can induce tremor, agitation, spasms and tics (20).

However, some facts suggest that the mechanism of action responsible for the antidepressant effect of SSRIs goes beyond the mere inhibition of 5-HT uptake and activation of 5-HT receptors (24). For example, it takes at least from 2 to 4 weeks for these drugs to exert an antidepressant effect, whereas the increased synaptic levels of monoamines are detected briefly after starting the treatment, suggesting that some adaptive changes must occur to achieve the desired effect. In addition, the acute depletion of tryptophan, the precursor of 5-HT, leading to a decrease in the brain levels of this neurotransmitter, does not induce a depressive behavior in healthy humans (25).

Still regarding the monoaminergic hypothesis, more recently DA was also reported to be implicated in the pathophysiology of depression and therefore suggested that DA uptake inhibition and dopaminergic neurotransmission potentiation may also be useful to achieve the antidepressant effect (26). In fact, the increased 5-HT_2*C*_ receptors activation favored by SSRIs can reduce the mean firing rate of the ventral tegmental area (VTA) neurons, leading to a decrease in DA that might account for reduced response to SSRIs (27, 28).

For this reason, triple reuptake inhibitors (TRI) combining enhancing properties for all the three monoamine neurotransmitters (5-HT, NE, and DA) have been postulated as good candidates to antidepressant drugs [see ref. (29) for a review] because, in addition to its broader target spectrum, some effects could compensate and attenuate side effects of selective reuptake inhibitors. For example, SSRIs induce hyperprolactinemia due to 5-HT-induced increase in prolactin (PRL) release causing sexual dysfunction, and this effect would be mitigated by increased DA (29). This would also explain the effects of monoamine oxidase (MAO) inhibitors, which potentiate monoamines inhibiting their metabolic degradation. This is the case of moclobemide, a MAO-A inhibitor which exerts antidepressant effects with lower sexual side effects than SSRIs (30). Unfortunately, the current clinical use of MAO inhibitors has considerably decreased because of safety concerns about the hypertensive reaction which can occur if tyramine-containing food (e.g., cheese) is eaten. Also, MAO inhibitors interact with other drugs which potentiate monoamines such as 5-HT reuptake inhibitors, TCAs and even opioid analgesics; or with adrenergic decongestants, leading to conditions such as serotonergic syndrome or hypertension (31). Moreover, MAO inhibitors are not promoted by major pharmaceutical companies and their clinical use is progressively disappearing as a content of medical residents training.

When designing TRIs, it is important to obtain an optimal relative inhibition of SERT, NET and DAT to avoid main side effects such as hypertension (high NET inhibition) or motor stimulation and stereotypies as well as abuse liability (excessive DAT blockade). For this reason, it has been suggested that an ideal TRI should have an *in vivo* ability to occupy a fraction of the population of monoamine transporters of SERT (≥80%) > NET (50–70%) > DAT (≤30%) (29), with a slow onset of DAT occupancy and slow clearance from brain to reduce abuse potential. According to these premises, numerous laboratories synthesized candidate compounds but, to date, none has reached the clinical market yet.

### Additional mechanisms

The discovery in the 1990s decade that antagonism at the NMDA glutamate receptors was capable of inducing antidepressant-like effects in mice exposed to the forced swim test (32) suggested that glutamatergic pathways may also play a role in depression. In fact, it is hypothesized that the pathology of major depression is associated with maladaptive activity of the intrinsic circuitry of the medial PFC (mPFC) and limbic system, where neurons predominately release glutamate and GABA rather than monoamines (33). Moreover, it was demonstrated that treatment with classical monoaminergic antidepressant drugs also induced adaptive changes on NMDA receptors and in brain areas important to mood control (34). This underpinned the involvement of a glutamatergic mechanism in the neurobiology of depression.

Accordingly, Berman et al. (35) published, at the beginning of 2000, a study showing that ketamine, a drug that was well known as a NMDA receptor antagonist, was able to induce fast (within hours) and lasting (up to 3 days) antidepressant effects with only one low dose. Since then, numerous clinical trials investigating the antidepressant effects of ketamine have been carried out (36) and some are still ongoing (37–39). The blockade of NMDA receptors by ketamine favors a series of events that are thought to be responsible for its antidepressant effect [see ref. (22) for a review], namely the reduced production of nitric oxide (NO), the increased presynaptic release of glutamate due to the decreased GABAergic inhibition, and the resulting increased activation of AMPA receptors with the subsequent activation of downstream pathways. The reduction of NO levels in CNS itself has been related with antidepressant effects as it can modulate the release of neurotransmitters and affect neuronal plasticity (40, 41). In addition, activation of AMPA receptors results in increased release of 5-HT which, activating postsynaptic receptors (especially 5-HT_1*A*_) in cortical regions, contributes to the antidepressant effect (42, 43), as it will be further developed below.

The increased AMPA receptor activation stimulates phosphatidylinositide 3-kinase (PI3K)/AKT–molecular target of rapamycin (mTOR) pathway both directly (through a calcium-dependent activation of kinases MEK/ERK and PI3K–AKT) and indirectly after inducing the opening of L-type voltage dependent calcium channels. This latter mechanism increases the release of brain-derived neurotrophic factor (BDNF) that activates the tyrosine kinase B (TrkB) receptors (44, 45) which, in turn, activate the PI3K–AKT-mTOR pathway, leading to increased neuroplasticity and neuronal survival. Neuroplasticity is, thus, essential for the antidepressant effect because inhibition of mTOR prevents the antidepressant effects of NMDA antagonists (46). In line with these findings, the neuroplasticity hypothesis of depression was developed (47). The ability of a drug to induce neuroplastic changes will therefore be a valuable property for a candidate to antidepressant drug, as will be discussed below. More recently (48) it has been demonstrated that antidepressant drugs can also directly bind to TrkB receptor dimers and stabilize the active conformation, thus enhancing neurogenesis.

Moreover, the inhibition of the glycogen synthase kinase-3β (GSK-3β) effects by drugs as ketamine or lithium, resulting in disinhibition of mTOR and reduced AMPA receptor internalization, is an additional mechanism which contributes to the antidepressant effect (49).

Several findings corroborate the role of neurogenesis in the pathophysiology of depression. For example, chronic stress impairs neurogenesis in rodents and humans, which improves after treatment with antidepressants for 2–3 weeks, a time matching their therapeutic latency (50–52). Antidepressants have been found to induce hippocampal neurogenesis and neuroplasticity in cellular and animal models of depression and improve the related behavior (52).

More recently, the ceramide pathway has also been suggested to be involved in depression (53, 54). Ceramide is formed from the membrane lipid sphingomyelin, after cleavage by acid sphingomyelinase (ASM). Ceramide and other bioactive sphingolipids have been found to act as signaling molecules in several cellular processes such as cell growth, death, inflammation, migration, adhesion and angiogenesis. Concretely, ceramide can be a cell death activator (55). The ASM/ceramide system can be activated by reactive oxygen species (ROS) and reactive nitrogen species (RNS) which, in the context of depression, are generated by endogenous sources, such as proinflammatory cytokines (e.g., IL-1, IL-6, TNFα) (56). Antidepressants such as desipramine, amitriptyline, fluoxetine, paroxetine, fluvoxamine or sertraline, among others, have been found to functionally inhibit ASM and, thus, the ceramide pathway. These drugs have in common to be lipophilic weak organic bases, with relatively low molecular weight, which can cross the plasma membrane and accumulate in lysosomes after being protonated. Consequently, they disturb the binding of ASM to the inner lysosomal membrane so that it is released to the cytoplasm, where it is inactivated by proteolysis (57). Moreover, reduction of ceramides in the hippocampus normalizes neurogenesis and behavior in mouse models of stress-induced depression (58). The reduction in ceramides is followed by a slow increase in sphingomyelin, which favors an increase in autophagy, which is also related with an improvement of depressive behavior (59, 60).

There is also a link between the effect of antidepressants on the ceramide pathway and the direct TrkB receptor activation. The dimerization of TrkB is enhanced by cholesterol and recent findings suggest that it is needed for the antidepressants to bind this receptor and stabilize the active conformation (59, 60). The cholesterol molecules in plasma membrane (near the TrkB receptor) can be displaced by ceramide, so the reduction of ceramide levels induced by antidepressants preserves membrane cholesterol, facilitating TrkB dimerization and further binding and potentiation by these drugs.

Overall, the studies on ketamine have brought new knowledge to understanding the neurobiology of depression and targets for new treatments. Some of the pharmacodynamic effects that this drug exerts can also be elicited by other drugs that recently have been proven to be effective in the treatment of psychiatric disorders (e.g., psychedelics), thus underpinning the role of these mechanisms. Ketamine and its FDA-approved enantiomer, esketamine, have demonstrated their efficacy and are usually well tolerated at the doses used as antidepressant. Although there may be some concerns about the appearance of psychotomimetic effects and other adverse reactions, these have been described only in a low percentage of patients or with very high doses that are administered for prolonged periods (61, 62). Therefore, these findings provide valuable information to consider in the development of new and improved antidepressant drugs (42, 43, 61).

Finally, another interesting point is that current evidence shows two ways to use drugs in the treatment of psychiatric disorders, namely administered as daily medication (e.g., classic antidepressants) or acutely as one or few doses accompanied by psychotherapy. Thanks to their underlying mechanisms of action, these drugs may help with neurocircuitry rewiring while modulating several neurochemical pathways. This could also assist with addressing negative emotional processing, which is characteristic of these conditions (62).

## 3,4-Methylenedioxy methamphetamine

Although it cannot strictly be considered an NPS, MDMA is one of the most representative examples of the use of a recreational illicit substance for therapeutic purposes. Although it had been synthesized by the pharmaceutical company Merck in 1912, MDMA never got officially to be tested as a therapeutical drug (63). In 1978, Alexander Shulgin, a Californian chemist, synthesized and tested the drug, being the first to describe that MDMA was a psychoactive drug in humans. MDMA induces changes in mood, lowering emotional defensiveness and increasing the ability to open to others, breaking boundaries in communication and increasing empathy and self-acceptance without producing marked hallucinogenic effects (64, 65). Therefore, it is not a “classic” psychedelic drug, but an “entactogen,” producing a gentler and easily tolerated state compared to LSD. Then MDMA was rescued and adopted by many underground therapists during the remaining 1970s and 1980s as an adjuvant of psychotherapy, reporting beneficial effects (66–68). Unfortunately, the enormous success that MDMA (also known as “ecstasy” and “Molly” in the street market) reached as a recreational drug due to its euphoric, pro-social and empathetic effects, led to its progressive placement in the most restrictive category of controlled substances worldwide. In spite of this, MDMA became one of the most widespread illegal drugs in the dance-club and rave party’s scenes, especially during the late 1980s and along 1990s, with numerous intoxication case reports and even some fatalities attributed to its consumption (69, 70).

However, after its scheduling and due to the previous evidence about its utility in psychotherapy, placebo-controlled studies conducted from the beginning of the 2000s provided preliminary safety and efficacy data on the use of MDMA in assisting psychotherapy for treatment-resistant PTSD (71–75). Later, phase 3 trials have been carried out to confirm the efficacy and safety to establish MDMA as a prescription medicine for the use in psychotherapy for PTSD (16). Approval could come as early as by the end of 2023.

In this phase 3 trial (16), patients with PTSD were subjected to three 8 h-experimental sessions of manualized psychotherapy spaced approximately 4 weeks, in which they received 80 mg MDMA orally, plus 40 mg after 1.5–2.5 h in the first session and 120 mg plus 60 mg in the second and third sessions. The comparison group received a placebo instead of MDMA, combined with the same psychotherapy. Each experimental session was followed by three 90-min integration sessions that were spaced ∼1 week apart to allow the participant to understand and incorporate their experience.

The patients were monitored for several vital constants (e.g., blood pressure, heart rate, body temperature, etc.) during the development of the sessions and, in general, systemic effects of MDMA were mild (transient systolic and diastolic blood pressure and heart rate, some reported muscle tightness, decreased appetite, nausea, hyperhidrosis, feeling cold…). Interestingly, no increase in adverse events related to suicidality was observed in the MDMA group.

The results demonstrate that these three doses of MDMA given in conjunction with manualized psychotherapy over the course of 18 weeks results in a significant and robust attenuation of PTSD symptoms and functional impairment, and that this attenuation is higher than in the placebo group. Moreover, the benefits seem to be bigger than the FDA-approved first-line pharmacotherapies sertraline and paroxetine, which have both exhibited smaller effect sizes in pivotal studies (76) and to whose many patients fail to respond. Long term assessments are warranted to evaluate the duration of the outcome.

MDMA is hypothesized to support and enhance psychotherapy by facilitating the ability of the subject to access emotionally upsetting material, modulating the associated level of arousal, and strengthening the therapeutic alliance (77). It has been suggested that “MDMA may catalyze therapeutic processing by allowing patients to stay emotionally engaged while revisiting traumatic experiences without becoming overwhelmed” (16).

MDMA possesses a unique combination of pharmacological properties that contribute to its overall psychotherapeutic effects [see ref. (78) for a review]. It is not the only monoamine releaser with particularly prominent effects on serotonin, but it also increases dopamine and noradrenaline and elevates serum oxytocin through activation of hypothalamic 5-HT_1*A*_ receptors (79, 80). Oxytocin is a neuropeptide believed to play a role in affiliation and bonding in mammals. However, the contribution of such increase in the entactogenic effect of MDMA is still a matter of controversy (78, 81). Mithoefer et al. (75) mentioned that MDMA may reopen an oxytocin-dependent critical period of neuroplasticity that typically closes after adolescence, thus facilitating the processing and release of particularly intractable, potentially developmental, fear-related memories. This, combined with therapy, may produce a “window of tolerance,” in which participants are able to revisit and process traumatic content without becoming overwhelmed or encumbered by hyperarousal and dissociative symptoms. Brain imaging studies found that activity in the amygdala, the brain structure that acquires and stores fearful memories (81), is reduced after MDMA administration, and shows changes in the response to angry and happy facial expressions (83, 84).

Also, cortisol concentrations significantly increase by 100–150% from baseline levels with MDMA ingestions of 0.5 and 2.5 mg/kg of body weight in the absence of physical exertion (85). These increased concentrations have been suggested to contribute to the fear extinction process when combined with psychotherapy (86).

Moreover, MDMA has also been demonstrated to induce neuroplasticity in rats across the corticolimbic circuitry. It stimulates the expression of the early gene transcription factor *c-fos* in the amygdala, hippocampus and cortex (87–89), which is an early marker of neuroplastic changes. Acute MDMA also raises brain-derived neurotrophic factor (BDNF) in cortical areas of rodents (90, 91) which leads to an increase in neurites and spines in cortical neurons (92, 93). MDMA can also induce neuroplasticity in the hippocampus of rats but, in this case, it requires chronic exposition (94). Neuroplasticity is necessary for the neurochemical rewiring which is supposed to account for the psychotherapeutic effect of this drug and other psychedelics, and this effect seems to be triggered after activation of 5-HT_2*A*_ receptors (95, 96).

Concerns on the clinical use of MDMA include potential side effects such as cardiovascular events, especially in elderly patients or those with previous cardiac conditions, and potential serotonergic neurotoxicity, although it has been reported that MDMA used under a clinical environment is unlikely to be neurotoxic (97, 98). Indeed, the results of the recent phase 3 trial (16) indicate that the MDMA regimen used in psychotherapy was not harmful in patients who did not meet the exclusion criteria, which included uncontrolled hypertension, history of arrhythmia, or marked baseline prolongation of QT and/or QTc interval. Under this controlled scenario, MDMA was safe and reported adverse effects were, in general, not severe.

Taken together, it can be stated that MDMA is one of the most evident examples that an NPS might, when used properly, be useful in psychiatry. It would also be a good starting point to design or screen substances that, having similar properties, present more favorable pharmacodynamics and less undesirable effects at therapeutic doses, as well as lack of neurotoxic effects after clinical use (98). The latter is fundamental for a drug to be approved for medical use by the regulatory authorities. In this line, MDMA analogs such as 3,4-methylenedioxyamphetamine (MDA) and 3,4-methylenedioxy-N-ethylamphetamine (MDEA) have also been explored for psychotherapeutic use (99–101). On the other hand, patients with a history of chronic MDMA consumption may not benefit from its therapeutic effects at the doses commonly used due to tolerance. Therefore, only drugs that do not have cross tolerance with MDMA could be used in these patients (78).

Finally, another field to explore is the stereochemistry, as isomers of the same compound can account for different effects. For example, in the case of MDEA, the S-isomer is responsible for its entactogenic effects, whereas the R-isomer produces dysphoria and depressive symptoms and is responsible for its neurotoxic effects (102).

## Cathinones

Synthetic cathinones, also known as β-ketoamphetamines, are a class of substituted amphetamines characterized by the presence of a carbonyl group at the β-position of the basic phenethylamine structure (see Figure 1 for representative chemical structures). Such modification approaches this structure to endogenous monoamines such as epinephrine and NE, which have a hydroxyl group at the β position. They are synthetic derivatives of cathinone, a natural alkaloid found in the shrub khat (*Catha edulis* Forsk), whose fresh leaves are traditionally chewed by population of the horn of Africa and the Arabian Peninsula to obtain stimulant and euphoric effects, as well to reduce hunger and fatigue and to ease social interaction (103, 104).

Synthetic cathinones are, together with synthetic cannabinoids, the most popular NPS and this correlates with the number of new substances appearing every year, although the prevalence of use of each individual substance is low compared with more classical drugs (105). Their consumption increased by the end of the first decade of 2000 when the production and purity of MDMA pills decreased due to the strong control on this drug and their synthesis precursors as well. However, not all the cathinones are NPS, as is the case of bupropion, which is a cathinone used as antidepressant and as an aid in smoking cessation, as will be mentioned below.

These compounds exert their effects targeting DAT, SERT and NET (106–115), mainly inhibiting monoamine uptake but some can act as substrates of the transporters and interact with the synaptic vesicles, inducing neurotransmitter release (106–115). These actions potentiate the effects of the monoamine/s involved and lead to strong psychostimulation. Also, depending on the serotonergic effects, some distortion of perceptions and entactogen effects can occur. For these reasons, synthetic cathinones are used as substitutes for amphetamines, MDMA or cocaine and have abuse potential. However, and according to the monoaminergic hypothesis of depression explained above, cathinones with an adequate profile of activity on the different transporters and lower addictive potential might also be candidates for antidepressant medications.

In fact, there is a published study carried out in mice assessing the acute antidepressant effects of the ethanolic extract of khat (116) showing a decrease in the immobility time in both the tail suspension test and the forced swim test. These results suggest antidepressant potential, at least in these animal models. However, other studies report schizophrenic-like symptoms in mice after subchronic oral administration of the extract (117) and the addictive potential of this natural drug is widely recognized (118), precluding any therapeutical use. Moreover, methcathinone was used in Russia as an antidepressant in the 1930s and 1940s although it was replaced by newer and safer drugs (119).

When looking for a possible therapeutic utility of existing cathinones, their pharmacological profile on different monoamine transporters must be carefully examined. The first generation cathinones mephedrone and methylone have DAT/SERT ratios between 0.1 and 10, which are higher than those obtained for MDMA (0.08) and in the range of that for cocaine (3.1) (115). High selectivity on DAT with > 10 DAT/SERT ratios may suggest increased abuse potential, especially given that DA has been related to reinforcing effects (120). For this reason, more favorable (lower) DAT/SERT ratios would be desirable for a cathinone to be considered as a therapeutic candidate, as it would also have predominant entactogenic effects over psychostimulant properties.

Bupropion, a dual DA and NE reuptake inhibitor and releaser, is a derivative of cathinone that was developed in the 1980s as an atypical antidepressant and has been prescribed in some cases of major depressive disorder (121) and as a smoking cessation agent. It has also been used to enhance the therapeutic response to noradrenergic and/or serotonergic antidepressants, decreasing sexual side effects and the weight gain induced by these drugs. It has negligible activity on SERT (IC_50_ > 10,000 nM) (122) and the fact that it is administered orally implies a pharmacokinetic profile that reduces its rewarding effects (123). However, abuse of bupropion through another administration routes (e.g., insufflation or injection of crushed bupropion tablets) has been described (124, 125).

Apart of considering the possibility that some specific cathinones, as bupropion, may be used in the chronic treatment of depression, some have been reviewed as potential candidates to be used as MDMA, namely in a few sessions, low doses and combined with psychotherapy because of their entactogenic effects. These included methylone, butylone and ethylone but, among them, methylone seems to better combine the desired properties (78).

Due to being the β-keto analog of MDMA, similar pharmacological effects would be expected for methylone, although noticeable differences have been reported. For example, *in vivo* 5-HT synthesis inhibition fully antagonizes the hyperlocomotion induced by MDMA but not that by methylone (107, 126). Methylone inhibits DAT and NET at approximately half the potency of MDMA and inhibits SERT at about one-third of its potency (107). However, unlike MDMA, methylone is not as potent inhibiting the vesicular monoamine transporters (VMAT2) at supposedly therapeutic concentrations (107, 127), which suggest lower releasing capabilities. On the other hand, methylone induces DA and NE release in rat brain synaptosomes and in HEK cells transfected with the human monoamine transporters with similar potency than MDMA, but it has lower potency for 5-HT release (111, 128, 129).

Methylone exhibits a lower potency than MDMA for 5-HT_2*A*_ receptors (130) and is a partial agonist at the 5-HT_1*A*_ receptor, with weak antagonist effect on 5-HT_2*C*_ receptors, where MDMA behaves as a partial agonist. However, other contemporary studies (115, 129) reported that methylone did not have significant affinity for any of these receptors.

In spite of everything, methylone consumers report sensations very similar to those experienced after taking MDMA, with subtle differences such as a shorter action, greater clarity of thought and a lower but noticeable increased desire to socialize and feelings of closeness to others. These perceptions suggest that it also has entactogenic effects (131) and that VMAT2 inhibition does not seem to be a requisite to induce such effects in humans (78). To note, these effects have been reported at doses of around 125 mg, which are around half the doses reported to induce some negative mood alterations (131).

A matter of concern about the therapeutic use of methylone may be the possible serotonergic neurotoxic effects reported from experiments in rats exposed to repeated doses [i.e., four doses of 25 mg/kg at 3-h intervals in 1 day (132) or 30 mg/kg, twice daily for 4 days (133)]. However, these effects were obtained after a binge dosing, which would not be the case if methylone was used therapeutically. In fact, MDMA has also been demonstrated to be neurotoxic after repeated administration (134) but, as mentioned above, the doses used in psychotherapy are far from these dosing regimens and unlikely to produce such deleterious actions. Moreover, Baumann et al. (111) examined post-mortem tissue concentrations of monoamines in rats following a schedule of a more moderate repeated dosing of mephedrone, methylone (three doses of 3 or 10 mg/kg every 2 h) and MDMA (three doses of 2.5 or 7.5 mg/kg every 2 h). In this case and in contrast to MDMA, neither mephedrone nor methylone depleted the monoamine content, which might represent a favorable aspect for methylone.

Overall, the most studied synthetic cathinones exert similar uptake inhibition at monoamine transporters as MDMA (127), but with slightly lower selectivity for SERT and overall lower potency (78). Moreover, they have very low potency inhibiting VMAT2 function, suggesting a scarce monoamine releasing effect and that the subjective entactogenic effects that some of them exert is due to a slightly different mechanism than MDMA (135). For this reason, such entactogenic effect deserves to be investigated to establish whether it can be useful to enhance the effects of psychotherapy on mood-related disorders. Also, designing drugs with a desired profile based on these previous findings is a promising option for obtaining new therapeutic compounds.

5-HT-releasing drugs have been used in the past as appetite suppressants and some are currently being evaluated for being used as anxiolytics or antidepressants (136, 137). In this line, synthetic cathinones displaying hybrid DA reuptake inhibition and 5-HT releasing activities have been suggested as potential therapeutic drugs (137, 138). This is feasible because certain chemical structures can bind to a given monoamine transporter (e.g., DAT) and block uptake whereas they can be substrate of another (e.g., SERT) and induce 5-HT release. Although some of such substances have already been synthesized and tested *in vitro* for such dual effects, *in vivo* experiments should confirm the appropriate potency and kinetics on every target to be tested for therapeutic purposes (138).

In this line, specific changes in the cathinone structure have been reported to change the mode of action on the monoaminergic targets to obtain drugs with the properties cited above. It is known that the addition of a 3,4-methylenedioxy group or substitutions on the phenyl ring, especially in para- position, of methcathinone generally shifts selectivity toward SERT and even can behave as partial 5-HT releasers (127, 128, 140–145). For example, it has been reported that trifluoromethyl-substitution of methcathinone in the para-position dramatically shifts the selectivity of methcathinone toward SERT (146), becoming a SERT-selective partial releaser. This effect has been suggested to be produced because these modified cathinones trap a fraction of SERTs in an inactive state by occupying a specific locus in the transporter called the S2-site. Moreover, these findings define a new mechanism of action for partial releasers, which is distinct from the other two previously known binding modes underlying partial release, such as that of MDMA analogs (acting on the S1-site) (147) or that of other phenethylamine derivatives, which trap the transporter in an inward facing conformation (148).

All this progress in the knowledge of monoamine transporters’ function and the structure-activity relationships concerning interaction with the different transporter binding sites can be the basis for the development of synthetic cathinones suitable for therapeutic use in psychiatry.

### Cathinones as a treatment for cocaine addiction

Specific cathinones have not only been suggested as antidepressants or aid in psychotherapy, but some may be useful to treat addiction to psychostimulants. Accordingly, bupropion analogs with a slower onset and longer duration of action on DAT compared with cocaine have been suggested as candidates for treating addiction to cocaine, methamphetamine, nicotine, and other drugs of abuse (149). Moreover, the fact that bupropion and other “atypical” DAT inhibitors such as benztropine and modafinil have been reported to be devoid of such a strong abuse liability profile as cocaine, has led to hypothesize that these drugs stabilize a DAT conformation which is different from that of cocaine-bound DAT (150). Concretely, experimental evidence led to suggest that cocaine and methylphenidate stabilize outward-open DAT conformation whereas the atypical inhibitors such as bupropion tend to be less affected by conformational changes and probably favor a more inward-facing occluded conformation which is responsible for the lack of addiction potential of these drugs (151).

Similarly, a pyrovalerone derivative, α-piperidinevalerophenone (α-PpVP), showed high affinity for DAT but very low potency inducing hyperlocomotion and place conditioning (106). Further research is needed to assess whether this is due to pharmacokinetic issues which prevent this drug from attaining sufficient concentration in brain, which, *a priori* is unlikely due to its high lipophilicity. Alternatively, the authors point out that α -PpVP could also act as an atypical DAT inhibitor due to the different binding mode observed in docking assays, which may trap the dopamine transporter protein in an atypical conformation. If it was the case, α-PpVP might also be suggested as a candidate aid for cocaine cessation.

On the other hand, S-mephedrone (S-MEPH), one of the two stereoisomers of mephedrone, has been reported to reduce anxiety- and depressant-like effects in cocaine- or MDPV-abstinent rats (152), suggesting that this compound may be a possible structural and pharmacological template to develop maintenance therapy to control the symptoms of withdrawal from psychostimulant abuse. In fact, the other stereoisomer, R-MEPH, is the main responsible for the rewarding effects of MEPH whereas S-MEPH is a 50-fold more potent 5-HT releaser than R-MEPH and does not induce place preference in rats.

## Psychedelics

Psychedelics are a class of hallucinogenic drugs whose primary effect is to trigger non-ordinary states of conciousness. This causes specific psychological, visual and auditory changes, and often a substantially altered state of conciousness. Apart from MDMA and ketamine, psychedelics constitute the main group of substances with ongoing clinical trials in search of new pharmacotherapies for mood disorders (153) (see Figure 1 for representative chemical structures).

The psychiatric effects of psychedelics are explained by their ability to bind and activate serotonergic receptors, which exist pre- and/or post-synaptically (154). Central serotonergic pathways, which originate from the raphe nuclei, are spread to most brain structures, especially the cortex, modulating the activity of a wide range of neurons (e.g., glutamatergic and dopaminergic) and many mood-related processes (155). Among the plethora of 5-HT receptor subtypes, 5-HT_2*A*_ is the main subtype attributed to be responsible for such effects, as it has been shown to be involved in reconsolidation of contextual fear, object recognition and conditioned food aversion memories, which are processes affected in psychiatric disorders such as depression, PTSD, anxiety and even drug addiction [see refs. (156, 157) as reviews]. Thereby, psychedelics may help with neurocircuitry rewiring addressing negative emotional processing and negativity bias, which can lead to more rumination, a common symptom of MDD (158).

The neurocircuitry rewiring induced by psychedelics is a complex process that is starting to be understood using functional magnetic resonance imaging (fMRI) (159–161). Psychedelics increase connectivity in high-level association cortices (partially overlapping with the default-mode, salience, and frontoparietal attention networks) and the thalamus while desynchronize and decrease what is called the “default-mode nertwork” connectivity (159, 160), which appears to be altered in mood diseases. This allows a new synchronous connectivity and rewiring responsible for the therapeutic effect. The cortical areas involved overlap with a map of 5-HT_2*A*_ receptor densities, which confirm its major role. The increase in global connectivity observed under psychedelics correlated with subjective reports of “ego dissolution” which is described as a sense of oneness with the universe or the experience of relaxed boundaries between the self and the world (162) and is related with a decrease in feelings of depression, anxiety, and stress, as well as with an increase in mindfulness-related capacities.

In fact, patients who participated in clinical trials assaying the effects of psychedelics on depression and reported amelioration of their disorder, related it as sense of connectedness to others (social connectedness), to the world and to past values, pleasant activities and hobbies, and felt more integrated, embodied and at peace with themselves and their troubled backgrounds (163).

Moreover, activity on 5-HT_1*A*_ receptor has also been reported to contribute to the therapeutic effects of psychedelics while counteracting some of the undesirable effects such as increased heart rate, vasomotor tone and blood pressure (164, 165). In fact, 5-HT_1*A*_ receptors have also been implicated in mood regulation (166) and are targets for drugs already approved for anxiety and depression such as buspirone or trazodone.

Below we will briefly review the psychedelics that have been investigated and used in the treatment of mood disorders, with the aim of extracting common characteristics required for new molecules which, keeping the therapeutic effects, might overcome some undesirable effects that may preclude their use in vulnerable patients.

### Psychedelics used in psychiatric disorders

#### Psilocybin/psilocin

Psilocybin is the most widely studied psychedelic nowadays, but the assays with this drug date back several decades, when Sandoz marketed and sold pure psilocybin to physicians and clinicians worldwide to use in psychedelic psychotherapy. Although the increasingly restrictive drug laws of the late 1960s curbed scientific research into the effects of psilocybin and other hallucinogens, its popularity as an entheogen (spirituality-enhancing agent) grew in the next decade, owing largely to the increased availability of information on how to cultivate psilocybin-containing mushrooms (167). The promising results from ulterior controlled clinical trials led to its placement as breakthrough therapy by the FDA for treatment-resistant depression in 2018 (168) and in major depressive disorder in 2019 (169), but it has also been postulated as a treatment for anxiety and substance use disorders (170). Recently published clinical trials (171) demonstrate that psilocybin exerts robust antidepressant effects (better than escitalopram) through global increases in brain network integration, as assessed by fMRI. Furthermore, psilocybin dampens the reactivity of the amygdala to negative stimuli (172) which, together with its neuroplasticity-inducing properties, gives sense to a possible use for treating fear-related disorders such as PTSD.

Psilocybin is a naturally occurring tryptamine found in several fungi genus, including *Psilocybe*, popularly known as “magic mushrooms,” and is responsible for its hallucinogenic effects. Upon oral ingestion, it is dephosphorylated in the liver into psilocin, its active metabolite, which crosses the blood-brain barrier to exert its psychedelic effects. These consist in altered perception with visual hallucinations and changes in mood, thought, cognition, and experience of self (173). It invokes profound and lasting changes in cognition, perception and emotion, and many consumers report a long-term improvement in their mental health after having used the drug (13, 174).

Psilocin exerts its effects mainly by activation of 5-HT_2*A*_ receptors, but it also binds to 5-HT_2*C*_ > 5-HT_1*A*_ > 5-HT_1*B*_ (in order of affinity). It also has moderate to lower affinity for other 5-HT receptors (e.g., 5-HT_1*B/D*_, 5-HT_5–7_), as well as for adrenergic α_2*A/B*_, DA D_3_ and imidazoline receptors (175). In addition, psilocin indirectly induces dopaminergic effects through stimulation of DA release in the caudate nucleus and putamen (176). The relative contribution of the effects on these receptors results in the overall effects of this drug. It has no relevant effect on SERT.

The low toxicity and addictive potential, and the fact that it is generally well tolerated makes psilocybin especially suitable for clinical use (177, 178).

#### *N*,*N*-Dimethyltryptamine/ayahuasca

*N*,*N*-Dimethyltryptamine (DMT) is a tryptamine derivative found in several plants and animals and is one of the main active molecules of ayahauasca. Ayahuasca is a plant beverage prepared by decoction from the Amazonian jungle vine *Banisteriopsis caapi* and the shrub *Psychotria viridis* [see ref. (179) for a review]. The first contains β-carbolines, especially harmine, tetrahydroharmine and harmaline (in traces), which are MAO-A inhibitors whereas the other contains DMT. β-carbolines, in addition to having an indirect monoaminergic effect, can also activate serotonergic receptors and inhibit the metabolism of DMT in the gut and the liver and thereby allow it to reach the CNS, exerting its psychedelic effects. Due to these properties, ayahuasca has long been used by indigenous groups from the Northwestern Amazon for ritual purposes. On the other hand, DMT has been detected endogenously in humans, and it has been suggested to play a role in the functioning of the CNS as it has been reported to be transported into the brain across the blood brain barrier (180).

Due to its resemblance with 5-HT, DMT interacts with many 5-HT receptor subtypes including 5-HT_1*A*_, 5-HT_1*B*_, 5-HT_1*D*_, 5-HT_2*A*_, 5-HT_2*B*_, 5-HT_2*C*_, 5-HT_5*A*_, 5-HT_6_ and 5-HT_7_ with varying degrees of affinity (181, 182). From these, we highlight again agonism at 5-HT_2*A*_ and HT_1*A*_ subtypes as preponderant in the psychiatric effects. DMT can also be substrate for the SERT and the vesicular monoamine transporter, so it can be stored in intracellular vesicles (183) and be transported into the nerve terminals. It has also affinity to trace amine receptors and been suggested to be a sigma-1 receptor modulator (180, 181) thus exerting complex regulatory effects on immunity and inflammation.

Several clinical trials have been performed using oral standardized ayahuasca extracts, demonstrating that acute administration of ayahuasca in the clinical setting to healthy volunteers is safe, and presents an acceptable tolerability [see refs. (179, 184) as reviews]. Moreover, improvements of mood disorders have been reported after its consumption even after a single dose (184–186).

Other modes of use include vaporization (smoking) of preparations containing higher doses of DMT to ensure reaching sufficient brain concentrations or intravenous or intramuscular injections to bypass the intestinal enzymatic breakdown. In both cases, the association with a MAO inhibitor is not necessary.

After consumption, initial effects of ayahuasca are typically of a stimulant-type sometimes accompanied by anxiety, elevated heart rate and blood pressure, confusion and a sense of being overwhelmed [reviewed by James et al. (184)]. This is followed by a vivid hallucinogenic experience, both colorfully visual and auditory, and even experimenting feelings of vibrations. Nausea and vomiting are the most frequently reported adverse effects found in the different studies.

#### 5-Methoxy-*N*,*N*-dimethyltryptamine

5-methoxy-*N*,*N*-dimethyltryptamine (5-MeO-DMT) is a naturally occurring tryptamine that can be found in leaves, bark and seeds of several plants in South American rainforest such as *Virola calophylla, Anandenantera peregrina* and *Dictyoloma incanescens*, among others. Other indole alkaloids such as DMT can also be found in such plants. Because of this composition, preparations from these plants have long been used as entheogens in rituals by indigenous populations of these areas. Also, 5-MeO-DMT is the main psychoactive component of the venom of the Sonoran Desert toad (*Bufo alvarius*) and can also been found in fungi such *Amanita citrina* and *Amanita porphyria* [see refs. (187) and (188) for reviews]. Moreover, it has been speculated that 5-MeO-DMT might also be endogenously produced in humans, although the existing reports are controversial (188).

An especial feature of 5-MeO-DMT is its binding profile, as it is a non-selective agonist of 5-HT receptors which binds to the 5-HT_1*A*_ receptors with higher affinity (K_*i*_ around 3 nM) compared to the 5-HT_2*A*_ receptors (K_*i*_ around 900 nM) (182, 189). However, as the authors of the study point out (189), the binding affinity (K_*i*_) to 5-HT_2*A*_ receptors must be taken with caution as these receptors exist in high- and low-affinity agonist binding conformations depending on whether they are coupled to G proteins. [^3^H]ketanserin is the 5-HT_2*A*_ antagonist radioligand used to label these receptors and labels both states with equal affinity, but the binding affinity of agonists for the 5-HT_2*A*_ receptor varies depending on whether the receptor is radiolabeled with an agonist or an antagonist, and agonists generally display 10-100-fold higher affinity for agonist-labeled 5-HT_2*A*_ receptors compared with antagonist-labeled receptors, as it was the case. Therefore, the binding affinity of 5-MeO-DMT for 5-HT_2*A*_ receptors might have been underestimated. In fact, a previous report investigating 5-HT_2*A*_ receptor activation in transfected SH-SY5Y cells reported an EC_50_ of about 300 nM (190). Moreover, 5-MeO-DMT is primarily inactivated through a deamination pathway mediated by MAO-A, and it is O-demethylated by cytochrome P450 2D6 (CYP2D6) enzyme to produce an active metabolite, bufotenine (191, 192), which binds with higher affinity to the 5-HT_2*A*_ receptor than 5-MeO-DMT (193) thus enhancing and prolonging the effects.

Also, binding of 5-MeO-DMT to SERT (182) and NET (189) with low micromolar affinity has been reported and may contribute to its action.

Human assays using either the inhaled dried toad secretion (194) or synthetic 5-MeO-DMT (inhaled or IM injection) (162, 195) have reported improvements in mood, mindfulness-related capacities, and life satisfaction 24 h after (or even sooner) a single administration, lasting up to 4 weeks after the intake, although a small proportion (2–10%) reported worsening. Another study carried out in a naturalistic group setting, ingestion of 5-MeO-DMT was associated with unintended improvements in depression, anxiety and stress (196).

The 5-MeO-DMT experience contrasts with the DMT experience, as the latter is known to produce particularly vivid and complex visual imagery rather than marked ego- dissolution (187). 5-MeO-DMT is a potentially useful addition to the psychedelic pharmacopeia because of its short duration of action, relative lack of visual effects and putatively higher rates of ego-dissolution and mystical experiences.

5-MeO-DMT is not devoid of adverse effects, which include fear, sadness, anxiety, confusion, fatigue, crying, paranoia, trembling, vomiting, nausea, headache, pressure on the chest or abdomen and loss of body perception [reviewed by Reckweg et al. (187)]. Subacute effects include flashbacks, i.e., short re-experiencing of some of the subjective 5-MeO-DMT effects and reactivations, i.e., brief (seconds) sensory disturbances such as flashes of light, especially during the week after 5-MeO-DMT exposure. Reactivations occur more frequently after vaporization as compared to intramuscular administration (195).

#### Lysergic acid diethylamide

Lysergic acid diethylamide is psychedelic drug of the ergoline family. As a difference with the other psychedelics exposed in this review, it is only obtained by hemisynthesis, and cannot be found in any natural source [see ref. (197) for a review].

LSD binds with nanomolar affinity to 5-HT_1*A*_, 5HT_2*A*_ and 5HT_2*C*_ receptors, as well as to dopamine D_2_ and adrenergic α_2_ and less potently to adrenergic α_1_, and dopamine D_1_, and D_3_ receptors (198). At 5-HT_2*A*_ receptors, LSD behaves as a partial agonist (175), which primarily mediate its hallucinogenic effects. In fact, LSD binds 5-HT_2*A*_ receptors more potently than other serotonergic psychedelics, which is a possible explanation to its stronger hallucinatory properties. Besides, LSD has also been proven to bind 5-HT_1*A*_ receptors more potently than other serotonergic psychedelics.

Before the FDA placed LSD in the Schedule I list in 1970, around a thousand clinical papers assaying LSD had been published, involving up to 40,000 patients in trials to treat addictions and other conditions (199). After, decades passed with barely any publications on the subject, until the 1990s, when interest into LSD resurfaced (197).

LSD is usually administered orally at doses in the order of μg (5 μg for a microdose to 400 μg for a heavy dose) being the most common doses around 150 μg. Its psychedelic effects start by 30 to 60 minutes post administration, peak at around two hours post administration and the effects stay high another 3–5 h lasting up to 12 h (200). However, LSD can also be administered intravenously, with the effects starting 15–30 min after administration and peak 45–90 min after dosing (201).

Physiologically, LSD induces an increase in blood pressure, heart rate and body temperature, as well as a plasma concentration increase in cortisol, prolactin, oxytocin and epinephrine (200). As it will be detailed below, such endocrine effects are also a common treat of psychedelics used in psychotherapy that has been linked to a part of their effects.

LSD can create tolerance. After 2–3 days of daily moderate doses, a tolerance is developed, and the same doses produce a decreased psychological effect. However, this tolerance disappears after 2–3 days of withdrawal, or placebo administration (202).

When receiving a dose of LSD in a clinical trial setting, participants experience auditory alterations, a feeling of well-being, visionary restructuralization (complex imagery coupled with audio-visual synesthesia during which visions change meanings) and oceanic boundlessness linked to depersonalization and derealization creating feelings of unity (200). Although these acute effects are very similar to those of other psychedelics, LSD perceptual alterations are reported to be stronger than those of other psychedelics. Generally, LSD is physiologically well tolerated and psychological reactions can be controlled in a medically supervised setting.

On the other hand, LSD creates an empathogenic mood similar to that experienced after taking MDMA. This empathogenic mood brings the participants feelings of closeness, openness and trust that enhance the efficiency of psychotherapy (200). LSD has also been reported to amplify the emotional response to music, an effect that is searched when used recreationally and that has been looked into to be used in therapeutic settings, as it has been noted that the combination of music and LSD might increase mystical experiences (203).

However, LSD has also been reported to induce anxiety through anxious ego-dissolution and disembodiment (200, 204), impaired cognition, cognitive disorganization, paranoia and fears of losing control, delusional thinking and flashbacks. These effects have informally been called “bad trip” (200) and are currently being treated to be reconducted to be valuable experiences for the patients (205). It is also frequent that the patients experience a combination of positive and negative sensations, with an overall valuation of positive experiences (204).

Also, flashbacks, or Hallucinogen Persisting Perception Disorder (HPPD), have been reported for LSD. This is an uncommon and poorly understood disorder in which individuals experience CNS malfunctions for up to a year after a single dose of a psychedelic drug, including visual distortions and hallucinations without being under the influence of a psychedelic drug (206). This is more commonly triggered in patients already diagnosed with a psychiatric disorder such as schizophrenia, or with a family history of such psychiatric illnesses, but can appear in healthy patients as well (206).

Furthermore, after a two-week follow-up, optimism and openness in most participants had increased and they felt heightened psychological wellbeing (204). This is supported by another study, that found improved mental wellbeing up to a year after a single dose of LSD (207).

LSD was tested in some randomized controlled trials as an aid in alcoholism treatment programs, and a meta-analysis of these studies reported that administration of a single dose of this drug was related with a decrease in alcohol misuse (208). On the other hand, the only randomized controlled trial that has been concluded to date with patients suffering from mood disorders is that performed by Gasser et al. (209). They administered either 200 μg or 20 μg of LSD (the second acting as an active placebo) during psychotherapy sessions to 12 patients suffering from anxiety related to life-threatening diseases. The results showed reductions in trait anxiety and state anxiety for up to a year post-treatment with no acute or lasting adverse effects starting at a day post administration (209).

After this successful trial, the Swiss government approved the compassionate use of LSD in a case-by-case basis (210).

Other non-completed clinical studies are investigating the effects of LSD on anxiety with volunteers with or without a life-threatening illness (ClinicalTrials.gov identifier 03153579).

### Mechanisms involved in the psychotherapeutic effects of psychedelics

#### Psychedelic-induced neuroplasticity

It is suggested that mood disorders such as MDD are consequence of a maladaptive neuronal plasticity and therefore drugs addressing this problem can be useful treatments. Nowadays, there is extensive evidence that psychedelics can rapidly induce neuroplasticity and correct the maladaptive process (211). This ability is shared with other new antidepressants such as ketamine, as demonstrated in a study using a synapse-targeting photoactivatable RAC1 (a small RhoGTPase). This study showed that the sustained antidepressant-like effects of ketamine in mice are mediated in part by spinogenesis in the prefrontal cortex (212). Moreover, the neuroplasticity induction by these drugs presents a rapid onset and is long-lasting and can be achieved after the administration of a single or multiple therapeutical doses (211).

Accordingly, activation of 5-HT_2*A*_ receptors by psychedelics induces an increase in extracellular glutamate in the prefrontal cortex which activates AMPA and NMDA receptors (213) leading to neuroplastic effects. These include increase in *c-fos* expression and early growth response gene 2 (*egr-2*). Studies with psilocybin demonstrated that these effects are abolished with a 5-HT_2*A*_ antagonist (93), underpinning that psychedelic-induced plasticity is mediated by these receptors. This ability to induce neuroplasticity is, again, a key property that makes this drug a candidate for treating fear-related disorders as the increased cortical activity could improve top-down regulation of the activity of the amygdala.

The neuroplastic effects of these compounds play a key role in their therapeutic effects, as they tend to restore the decreased neuroplasticity and neural atrophy in the PFC (214–216), which has been described as a common feature of the psychiatric diseases for which they are used (217–220). Due to this common mechanism and pharmacological effect, drugs capable of inducing neuroplasticity in PFC, as serotonergic psychedelics, constitute a general option to treat all this sort of disorders (93).

Brain-derived neurotrophic factor (BDNF) is a key neuroplasticity-modulating neurotrophin that is involved in many physiological and pathological brain processes (221, 222). It has been reported that psychedelics and related compounds, inducing the activation of AMPA and NMDA receptors, can positively modulate the levels of BDNF and/or neurotrophins which, inducing neuroplasticity, would ameliorate disorders originating from altered neuronal connectivity (223). For example, a single, low dose of LSD acutely increases BDNF levels in healthy volunteers (224). Similarly, a double-blind randomized placebo-controlled clinical trial reported that a single dose of ayahuasca could modulate serum BDNF in patients with major depressive disorder which agrees with the previously reported antidepressant effects of psychedelic brew (225). The primary psychoactive component of ayahuasca is DMT, so it is likely that 5-MeO-DMT, which is a very close structural analog of it, also exerts effects on BDNF and promotes neuroplasticity in humans.

Psychedelic-induced neuroplasticity includes increased neurogenesis, spinogenesis, and synaptogenesis, as shown from in both *in vivo* and *vitro* models (93). Among several beneficial consequences of these processes, it has been suggested that the increased synaptic plasticity may reverse the stress-induced changes in prefrontal cortex and contribute to the antidepressant action of psilocybin in PTSD (226).

#### Antiinflammatory effects

The activation of 5-HT_2*A*_ receptors also exerts anti-inflammatory effects that would help to control the neuroinflammation associated with mood disorders (227–229). More concretely, activation of 5-HT_2*A*_ receptors blocks the generation of the pro-inflammatory factors TNF-α and IL-1β and reduces the levels of IL-6 and cyclooxygenase 2 (COX-2).

Moreover, interaction of these substances with sigma-1 receptors has also been suggested to play a key role in the protection from neuroinflammation and in neurogenesis. Sigma-1 receptors modulate neuronal differentiation, can act as intracellular signal transduction amplifiers, protect cells against reactive oxygen species, modulate inflammation, regulate BDNF secretion and can inhibit apoptosis signaling (230–233). For these reasons, compounds with agonist properties at sigma-1 receptors show antidepressant effects and are also effective in complications of depression (234, 235). Although it did not involve psychedelics, the recently published results of a clinical trial with a combination of dextromethorphan plus bupropion report that this combination significantly improved depressive symptoms compared with bupropion and was generally well tolerated (236). In this case, dextromethorphan combines sigma-1 receptor agonist and uncompetitive NMDA receptor antagonist properties. Moreover, dextromethorphan has been reported to interact with and inhibit NET at low micromolar concentrations (237–239), which might also contribute to the antidepressant effect. On the other hand, bupropion increases dextromethorphan plasma concentrations by inhibiting its metabolism (236) and may contribute to the overall antidepressant effect as well.

#### Endocrine effects

Some of the studies investigating the therapeutical use of psychedelics also examined their effects in mood-related hormones. One of these hormones is oxytocin, whose intranasal administration has been tested in psychiatry for anxiety, depression and PTSD (240). Individual assays demonstrate beneficial effects on the studied disorders, although when all these studies are reviewed together it has been reported that the actual efficacy of oxytocin is still inconclusive (240). However, it has been suggested that there is a correlation in the methylation of the promoter of the oxytocin receptor gene, leading to reduced response to oxytocin, and the severity of depression (241). Moreover, a recent meta-analysis (242) reported altered endogenous oxytocin concentrations in several psychiatric disorders compared with controls, although there were no significant changes in depression and PTSD was not analyzed. Nevertheless, another contemporary study showed that the oxytocin level was inversely related with depressive symptomatology in women (243).

As cited above, increased oxytocin levels have been suggested to contribute to the entactogenic effects of MDMA (80), thus enhancing the effectivity of psychotherapy. Similarly, LSD, at a dose of 200 μg, moderately increases plasma oxytocin levels compared with placebo and dose-dependently (25–200 μg) increases implicit and explicit emotional empathy (244). The oxytocin increase was prevented by the 5-HT_2*A*_ antagonist ketanserin, which demonstrates the direct implication of these receptors. Another study confirmed this effect for LSD and compared its effects with psilocybin, reporting that both drugs significantly increase oxytocin levels and plasma cortisol and prolactin as well (245).

We could not find any article assessing the effects of ayahuasca or DMT on oxytocin levels. Instead, it has been reported that ayahuasca (which contains a mixture of IMAOs and DMT) induces increases in plasma levels of prolactin, cortisol, and growth hormone (246). These effects may be the consequence of the mixed actions of this brew due to the intense MAO inhibition, leading to potentiation of other monoamines in addition to 5-HT. Although we could not find reports on DMT endocrine effects it may be hypothesized that, as it is a 5-HT_2*A*_ agonist as LSD and psylocibin, it also might increase oxytocin levels.

Another affected hormone can be prolactin (PRL). Ayahuasca increases blood cortisol and prolactin (PRL) levels (247, 248) and 5-MeO-DMT also strongly promotes the release of PRL in rats (249) which is followed by a decrease and is consequence of hypothalamic 5-HT receptors activation, resulting in systemic anti-inflammatory and immunomodulatory effects through prolactin receptor-expressing immune cell types (187). Furthermore, such increases were higher than those induced by its tryptamine analogs, bufotenin and DMT.

## Concluding remarks: Clues for obtaining new psychotherapeutic compounds inspired by cathinones and psychedelics

**FIGURE 2:**
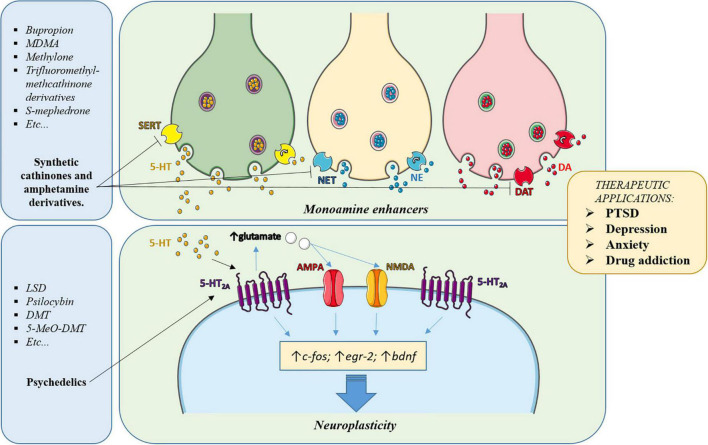
Simplified scheme of the main pathways involved in the psychotherapeutic effects of the drugs subject of this article. Classic antidepressants, MDMA and cathinones enhance the synaptic levels of monoamines (5-HT, DA, NE) in key brain areas, leading to activation and regulation of the levels of their receptors, thereby contributing to normalizing the brain circuitry involved in the disorder. Also, the increased synaptic levels of 5-HT induced by these drugs or the direct agonism exerted by psychedelics, activate serotonergic receptors, especially the 5-HT_2*A*_ subtype, whose activation triggers intracellular pathways involved in neuroplasticity. In addition, activation of 5-HT_2*A*_ receptors induces glutamate release in cortical areas, which activates AMPA and NMDA receptors, further contributing to neuroplasticity and rearrangement of the altered connectivity in the affected brain areas.

In this article we have reviewed the properties of drugs useful to treat psychiatric disorders and we have highlighted the properties they share and that seem to be responsible for their therapeutic effects. Some of them had been initially used for recreational or ceremonial purposes but were reconducted to a clinical setting. As cited in the introduction, the continuous appearance of NPS, especially from the cathinone and the tryptamine families, can provide new chemical structures having the right combination of the desired properties. Those accomplishing these requirements may be tested in a clinical setting as new candidates for treating those disorders. It can also be highlighted that, although differing in their principal target, most of these drugs, directly or indirectly converge in the modulation of intracellular pathways contributing to the therapeutic effects (Figure 2).

There is no doubt that activation of 5-HT_2*A*_ receptors is an important requirement, either directly or indirectly (i.e., inducing 5-HT release) which is necessary for the neuroplastic properties and other effects, but there is evidence that, through molecular modeling, the affinity and neuroplastic properties of a molecule can be kept whereas hallucinogenic and other adverse effects may be removed.

For example, Cameron et al. (250), through careful chemical design, modified ibogaine (a non- conformationally restricted analog of 5-MeO-DMT, to a safer, non-hallucinogenic psychedelic compound variant (called TBG) with therapeutic potential for treating drug addiction. Most 5-HT_2*A*_ agonists also have activity on 5-HT_2*B*_ receptors, which can lead to cardiac valvulopathy (251) but, in this case, the compound TBG behaved as an antagonist on these receptors, thus bypassing this potential adverse effect. This is also an example that selectivity for the 5-HT_2*A*_ in front of other subtypes can be enhanced. The study of the pharmacological properties of the stereoisomers of a candidate molecule may also provide valuable information for enhanced potency and/or selectivity, as in the case of MDMA (102).

Nevertheless the 5-HT_2*A*_ receptor activation by the drug may be optimized in terms of potency and duration, in order to avoid excessive and negative sensations while keeping the beneficial effect. Also, pharmacokinetics can be improved by modifying properties such as lipophilicity and rapid metabolic inactivation. New tryptamine derivatives are continuously appearing in the NPS market and detected in consumers (252, 253) so their pharmacological properties should be studied to assess and provide information about their characteristics and about a possible therapeutic use in a clinical setting.

Also, bearing mild dopaminergic properties without exerting reinforcing effects may help to the therapeutic effect as occurs with classical antidepressants. On the other hand, affinity for sigma-1 receptors is another interesting property that is shared among these molecules and that has been suggested to be involved in the intracellular signaling processes in which they are involved (230). To note, several compounds with NMDA receptor antagonist properties have also affinity for sigma-1 receptors (e.g., dextromethorphan), both properties reported to contribute to the antidepressant effect.

Another interesting aspect that is being investigated is psychedelic microdosing. This consists in administering a 10th, or even less, of the usual therapeutic dose. Studies in rats report that chronic, intermittent, low doses of DMT produce antidepressant-like effects and enhanced fear extinction, without affecting working memory or social interaction (254). Also, recent studies in humans using two to four microdoses of LSD or psilocybin (255, 256) report an increase in positive mood, a decrease in depression, augmented energy, and improved work effectiveness. However, more research using double-blind placebo-controlled trials is needed to really confirm the efficacy of this dosing approach.

Finally, all the exposed above justifies the need to study, at different levels, the properties and effects of NPS as some of them may provide new therapeutic tools for psychiatric disorders.

## Author contributions

DP, RL-A, and EE contributed to the conception and design of the study. DP wrote the manuscript draft. RL-A made the figures. All authors provided contents and references to be included in the review and critically read the manuscript and approved the submitted version.

## Footnotes

1
https://maps.org/
